# Barriers to antenatal care use in Nigeria: evidences from non-users and implications for maternal health programming

**DOI:** 10.1186/s12884-015-0527-y

**Published:** 2015-04-17

**Authors:** Adeniyi Francis Fagbamigbe, Erhabor Sunday Idemudia

**Affiliations:** School of Research and Postgraduate Studies (SoRPS), Faculty of Human and Social Sciences, North West University, Mafikeng, South Africa; Department of Epidemiology and Medical Statistics, Faculty of Public Health, College of Medicine, University of Ibadan, Ibadan, Nigeria

**Keywords:** Antenatal care, Nigeria, Wealth, Education, Barriers to health care utilization

## Abstract

**Background:**

In Nigeria, over one third of pregnant women do not attend Antenatal Care (ANC) service during pregnancy. This study evaluated barriers to the use of ANC services in Nigeria from the perspective of non-users.

**Methods:**

Records of the 2199 (34.9%) respondents who did not use ANC among the 6299 women of childbearing age who had at least one child within five years preceding the 2012 National HIV/AIDS and Reproductive Health Survey (NARHS Plus II), were used for this analysis. The barriers reported for not visiting any ANC provider were assessed vis-à-vis respondents’ social demographic characteristics, using multiple response data analysis techniques and Pearson chi-square test at 5% significance level.

**Results:**

Of the mothers who did not use ANC during five years preceding the survey, rural dwellers were the majority (82.5%) and 57.3% had no formal education. Most non-users (96.5%) were employed while 93.0% were currently married. North East with 51.5% was the geographical zone with highest number of non-users compared with 14.3% from the South East. Some respondents with higher education (2.0%) and also in the wealthiest quintiles (4.2%) did not use ANC. The reasons for non-use of ANC varied significantly with respondents’ wealth status, educational attainment, residence, geographical locations, age and marital status. Over half (56.4%) of the non-users reported having a problem with getting money to use ANC services while 44.1% claimed they did not attend ANC due to unavailability of transport facilities. The three leading problems: “getting money to go”, “Farness of ANC service providers” and “unavailability of transport” constituted 44.3% of all barriers. Elimination of these three problems could increase ANC coverage in Nigeria by over 15%.

**Conclusion:**

Non-use of ANC was commonest among the poor, rural, currently married, less educated respondents from Northern Nigeria especially the North East zone. Affordability, availability and accessibility of ANC providers are the hurdles to ANC utilization in Nigeria. Addressing financial and other barriers to ANC use, quality improvement of ANC services to increase women’s satisfaction and utilization and ensuring maximal contacts among women, society, and ANC providers are surest ways to increasing ANC coverage in Nigeria.

## Background

Antenatal care (ANC) is the care a pregnant woman receives during her pregnancy through a series of consultations with trained health care workers such as midwives, nurses, and sometimes a doctor who specializes in pregnancy and birth [1-3]. An analytical review of the recent World Health Statistics showed that ANC coverage, between 2006 and 2013, was indirectly correlated with maternal mortality ratio (MMR) worldwide. This indicates that countries with low ANC coverage are the countries with very high MMR [2,4-7]. For instance, ANC coverage in United Arab Emirates was 100% with MMR of 8 per 100,000 and Ukraine had 99% ANC coverage and MMR of 23. By comparison, in sub-Saharan Africa, Ghana had ANC coverage of 96% and MMR of 380/100000, Chad had 43% ANC coverage and a MMR of 980/100,000, and Nigeria had ANC coverage of 61% and MMR of over 560. Nigeria’s MMR is clearly above the African and global average of 500 and 210 respectively [8]. The poor maternal health outcome in Nigeria could be a result of poor ANC utilization [6,9] although ANC coverage may not provide information on the quality of care provided [10].

The importance of ANC services in the outcomes for pregnant women has been well documented [2,5,11,12]. ANC enhances early identification and management of conditions that could be threatening to the mother and her unborn child. ANC by trained skilled providers screens for infections, treats malaria, reduces the incidence of perinatal illness and death, provides birth preparedness, identifies signs of danger in pregnancy and plans to handle possible delivery complications through timely treatment and referrals [2,5]. It also reduces medical problems in pregnancy such as anaemia, hypertension, ectopic pregnancy, obstructed labour, eclampsia, excessive bleeding and premature labour and delivery [5,10,11,13,14]. In particular, a clinical audit of antenatal services in Nigeria found better maternal outcomes among women who had completed ANC than those who had not [5] though it may not directly reduce the risk of death [15].

Two nationally representative surveys were conducted recently in Nigeria: Nigeria Demographic and Health Survey (NDHS) in 2013 and National AIDS and Reproductive household survey (NARHS) in 2012 [1,3]. The two surveys showed that the proportion of pregnant women who had not attended any ANC services in Nigeria was 33.9% and 34.9% respectively. According to the 2013 NDHS, only 60.9% among women of child bearing age (15–49 years) who had a live birth in the five years preceding the survey received ANC from a trained skilled ANC provider (i.e., a doctor, nurse or midwife, or auxiliary nurse or midwife). Only half (51.0%) reported making four or more ANC visits during the pregnancy. About one third (36%) of births were delivered in a health facility while 38% of all deliveries within the five years were assisted by a skilled birth assistant (SBA) [1,3]. The attendance of ANC and delivery in a facility by a trained birth assistant are far lower than most other Africa countries [8,16]. In sub-Saharan Africa, overall 75% had at least one ANC attendance, 48% had 4 or more ANC visits and 48% of deliveries were supported by skilled birth attendants [8,17].

In comparison with ANC coverage in Nigeria, a neighboring developing country, Mali, had 57% of pregnant women having at least one prenatal contact with a skilled ANC provider within five years preceding the DHS in 2001 [18,19]. In another developing country, Indonesia, about 95% of pregnant women attended at least one ANC visit and 66% of women had four ANC visits within five years before the 2007 DHS [20]. This implies that Nigeria has not attained maternal health care success achieved over a decade ago in Mali and over 5 years ago in Indonesia. The questions are why are pregnant women not attending ANC in Nigeria? What are the limiting factors? What are the barriers?

Studies have documented the socio-demographic and other factors affecting ANC use. Lincentto et al. identified inability to pay for ANC services or prescribed treatment as an important barrier to utilization of ANC [2], a finding supported by two other studies [20,21]. In situations where ANC uptake requires travel and long waiting hours, pregnant women and their families experience huge opportunity costs, such as the loss of income in order to attend services [2,22]. Long distances to health facilities as well as insufficient number of ANC providers at various ANC clinics negatively affect ANC utilization [23].

Several studies have identified rural–urban differentials in use of ANC in Nigeria and elsewhere [2,6,9,21,23-25]. The higher ANC coverage in urban areas than in rural areas worldwide has been ascribed to inequities in the number of accessible health facilities [12,26]. In Nigeria, urban bias in public health expenditure, inadequate financing coupled with difficulties in attracting health workers to and retaining them in rural areas have limited government’s ability to create an accessible community-based health care system [26] which could reduce inequities in rural–urban health facilities. This scenario also occurs in other developing countries [25].

Family members of pregnant women as well as the community, have roles to play in ANC attendance. Their involvement in ANC utilization or otherwise affects use of ANC services. Families and communities often consider pregnancy as a natural process of life and therefore, underestimate the importance of ANC [2]. Misunderstandings, conflict or poor communication among formal and informal health care providers and with health service seekers may cause low utilization of ANC services in certain communities [2]. Unprofessional practices, attitudes and behaviours of ANC providers may further increase the non-utilization of ANC. Unprofessional conduct may include failure to respect the privacy, confidentiality, and traditional beliefs of the health seekers, [9,23].

There is a need to understand the reasons and in particular the limiting factors for the low rates of ANC uptake and by extension birth deliveries by a skilled provider in Nigeria. While several studies have identified determinants and factors affecting ANC utilization, very few have focused on documenting the barriers to ANC utilization from the perspectives of the non-users. This is probably due to the difficulties in collecting meaningful data from a reasonably large number of non–users. The current study used data from over 2000 ANC non-users. Analysis is designed to identify and prioritize reasons given by respondents for not utilizing ANC facilities in Nigeria.

## Methods

### Data source

We used the data from the 2012 National HIV/AIDS and Reproductive Health Survey (NARHS Plus II), [1] a cross-sectional study covering sampled men and women of reproductive age. Respondents were selected from rural and urban areas in all 36 states and the Federal Capital Territory (FCT) in Nigeria, as contained in the updated sample frame developed and maintained by the National Population Commission (NPC).

Four-stage cluster sampling was used to select eligible persons. Selection of rural and urban localities from each state and the FCT at stage 1; at stage 2, Enumeration Areas (EA) within the selected rural and urban localities were selected; households were listed at stage 3 while stage 4 involved selection of individual respondents for interview. Thirty two individuals were sampled from each of the 30 sampled EA (clusters) from each state. Overall, 35,520 individual respondents were selected for final interview of which 31,235 individuals (88%) were successfully interviewed [1]. Other details of sampling methodology have been reported [1].The Institutional Review Board (IRB) of the National Institute of Medical Research, Nigeria granted ethical clearance to the survey instruments and materials prior to the commencement of the survey. Details of the ethical approvals have been reported earlier [1].

Of the 15567 women interviewed in the 2012 NARHS, only 6299 reported to have had at least one child within five years preceding the survey. About one third (34.9%), 2199, of the 6299 mothers declared not have visited any ANC providing facilities before. All analyses in this paper were based on the responses of these 2199 respondents.

### Variables

The outcomes of interest in this study were the proportions of women who gave various reasons for not attending ANC during their last pregnancy. They were to answer “Yes” or “No” to reasons the possible reasons why they did not attend ANC. A “Yes” response implied “A big problem” while a “No” response implied “Not a problem”. The assessed reasons were economic, social, cultural, individual, family and ANC service provider related. They include “Obtaining permission from Spouse”, “Obtaining permission from Guardian/Parent”, “Obtaining permission from Others”, “Getting money to go”, “Far distance from health facility”, “Availability of transport”, “Need an accompany/Can’t go alone”, “Provider is not of the same sex”, “No skilled health worker in the hospital/clinic”, “Poor attitude of the health provider”, “Can’t guarantee confidentiality of information” and “Poor availability of good drugs at the facility”.

We then compared the reasons given among the socio-demographic characteristics which included: “wealth status - poorest, poorer, Average, wealthier and wealthiest”, “educational attainment - no formal education, primary, secondary and higher (tertiary)”, “marital status – currently married, formerly married and never married”, “location of residence – urban and rural”, “geo-political zones – North Central, North East, North West, South East, South South and South West”, “age of respondent at birth was recoded into <20 years, 20–24 years, 25–34 years and 35–49 years”, “tribe – Hausa/Fulani, Igbo and Yoruba”, “religion – Islam, Christianity and Others”, “employment status – Employed and Unemployed”. For all the variables, 5 responses given as “Don’t Know” were excluded from further analysis.

### Statistical analyses

Due to the multistage cluster sampling technique used in the random selection of the sampling units, we weighted the data by introducing a weighting variable. This was aimed at ensuring that the sample reflected population differences across the states and also avert over sampling of hard to reach areas and under sampling of large populations. The intra-cluster correlation was minimized through the use of effective sample size.

We used descriptive statistics to describe the distribution of the socio-demographic and behavioral characteristics of the respondents not using ANC. Bivariate analyses of relationships between the characteristics and reasons given for not taking ANC services were conducted using Pearson Chi-square (X^2^) test of association. Multiple response data analysis techniques were used to identify and prioritize the multiple reasons given by respondents for not attending ANC clinics. Multiple responses arise when more than one response may be given by the respondents to one question. Details on methodologies for handling the problem of multiple responses have been documented [27]. All statistical tests were performed at 5% significance level. We used STATA 13 and SPSS IBM 20 to analyze the data.

## Results

The mean age of the respondents who did not use the ANC service was 29.1 ± 8.2 years and was not significantly different from age of respondents (29.4 ± 6.7 years) who utilized ANC (p > 0.05, not shown in the tables). In Table 1, rural dwellers were the majority (82.5%) of the mothers who did not use ANC during the period covered, 57.3% of them had no education and nearly all the non-users (93.0%) were either currently married or living with sexual partners. North East was the geographical zone in Nigeria with highest number of non-users as they had 42.1% compared with 3.6% in the South East. Nearly all, 96.5% of them, were employed while 50.6% were of Hausa/Fulani tribe. Also 43.3% of mothers in rural areas did not use ANC compared to 18.2% in the urban area 61.7% among mothers in the poorest wealth quintile did not use ANC compared to 8.0% among mothers in the wealthiest quintile.

**Table 1:** **Distribution of Socio-demographic characteristics of the users and non-users of ANC facilities**

**Characteristics**	N (%)*	**Used ANC**	**Didn’t use ANC**	**N^**	%**
Location					
Urban	2112 (33.5)	81.8	18.2	383	17.5
Rural	4187 (66.5)	56.7	43.3	1816	82.5
Education					
No formal Education	2105 (33.4)	40.3	59.7	1259	57.3
Qur’anic Only	523 (8.3)	56.4	43.6	228	10.4
Primary	1126 (17.9)	71.0	29.0	327	14.9
Secondary	2020 (32.1)	83.1	16.9	341	15.5
Higher	521 (8.3)	91.6	8.4	44	2.0
Marital Status					
CM/LWSP	5883 (93.9)	65.6	34.4	2027	93.0
Form Married	221 (3.5)	57.0	43.0	95	4.4
Never Married	159 (2.5)	63.5	36.5	58	2.6
Age At Birth					
<20	371 (5.9)	50.4	49.6	184	8.3
20-24	1228 (19.5)	60.8	39.2	481	21.9
25-34	3091 (49.1)	69.8	30.2	934	42.5
35-49	1610 (25.6)	62.8	37.2	600	27.3
Wealth Quintiles+					
Poorest	1495 (23.7)	38.3	61.7	923	42.0
Poorer	1359 (21.6)	51.8	48.2	655	29.8
Average	1166 (18.5)	72.8	27.2	317	14.4
Wealthier	1127 (17.9)	81.5	18.5	209	9.5
Wealthiest	1150 (18.3)	92.0	8.0	92	4.2
Zone					
North Central	936 (14.9)	66.0	34.0	318	14.5
North East	773 (12.3)	50.7	49.3	381	17.3
North West	1797 (28.5)	48.5	51.5	925	42.1
South East	537 (8.5)	85.7	14.3	78	3.6
South South	899 (14.3)	69.2	30.8	277	12.6
South West	1358 (21.6)	83.8	16.2	220	10.0
Tribe					
Hausa/Fulani	2147 (34.1)	48.2	51.8	1113	50.6
Igbo	761 (12.1)	86.7	13.3	102	4.6
Yoruba	1192 (18.9)	84.6	15.4	183	8.3
Others	2199 (34.9)	63.6	36.4	801	36.4
Religion					
Islam	3241 (51.4)	55.6	44.4	1437	65.4
Christian	2995 (47.6)	75.9	24.1	725	32.9
Others	63 (1.0)	41.3	58.7	37	1.7
Employment					
Employed	6044 (95.9)	64.9	35.1	2122	96.5
Unemployed	256 (4.1)	70.0	30.0	77	3.5
Total	6234 (100)	65.2	34.8	2199	100

CM/LWSP Currently married or Living with sexual partner + some responses were missing.

*Distribution of all women who were pregnant within 5 years preceding the survey.

**Distribution of women who did not use ANC during the period.

^number of women who didn’t use ANC during the period.

**Figure 1:**
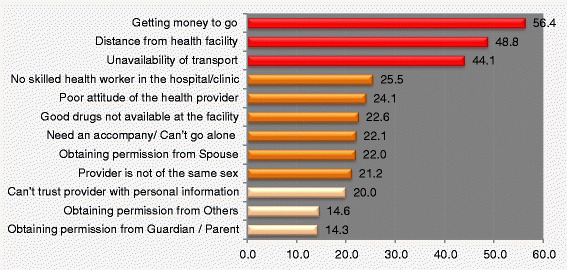
Distributions of reasons for not attending ANC.

As shown in Table 2 and Figure 1, the problem of getting permission from the respondents’ spouses to attend ANC were cited by 22.0% of the respondents, 14.3% had a problem getting permission from parents/guardian and 14.6% had a problem in getting permission from other family members, cultural or religious leaders. Over half (56.4%) of the non-users reported having a problem with getting money to go for the ANC services while 44.1% claimed that they did not go because transport facilities to the service providers were not available. The reasons given were significantly associated with most socio-demographic characteristics of the respondents except employment status. However, the problem of getting money to go for ANC services was not significantly associated with mothers’ age or current marital status.

**Table 2:** **Distribution of permission reasons for not utilizing ANC services by respondents’ socio-demographic characteristics**

	**Obtaining permission from spouse**	**Obtaining permission from parent or guardian**	**Obtaining permission from others**	**Getting money to go**	**Unavailability of transport**	**Need accompaniment/ cannot go alone**
Location						
Urban	*15.9	*10.7	*11.3	*40.3	*29.6	*16.2
Rural	23.4	15.0	15.4	59.8	47.2	23.7
Education						
No Formal Ed	*26.5	*17.9	*18.9	*58.6	*48.3	*26.9
Qur’anic Only	23.7	15.8	12.8	56.8	47.8	22.9
Primary	16.4	8.6	9.6	55.9	40.1	16.7
Secondary	11.9	6.5	6.8	50.0	30.5	10.9
Higher	7.1	4.8	2.4	42.9	42.9	21.4
Marital Status						
CM/LWSP	*23.1	*14.8	*15.3	56.2	44.7	22.7
Form Married	8.4	3.2	8.3	61.1	40.0	24.2
Never Married	8.8	15.8	5.3	61.4	36.8	10.5
Age At Birth						
<20	*24.7	*19.8	*16.5	57.1	*51.1	*29.5
20-24	25.2	15.0	15.0	58.7	47.8	22.9
25-34	22.3	15.0	15.6	58.1	44.6	23.6
35-49	18.3	10.8	12.3	51.7	38.4	17.8
Wealth Quintiles						
Poorest	*28.0	*17.9	*18.8	*63.1	*53.6	*29.0
Poorer	20.7	15.7	15.7	57.8	43.6	21.0
Average	17.8	9.9	9.6	52.4	34.5	16.2
Wealthier	12.5	3.4	5.8	41.3	30.3	11.1
Wealthiest	8.8	6.6	3.3	27.2	18.7	13.2
Zone						
North Central	*19.1	*9.9	*9.5	*62.7	*47.8	*16.6
North East	25.7	15.5	17.1	66.1	58.5	31.2
North West	27.9	21.2	21.6	54.2	43.8	27.7
South East	6.4	1.3	0.0	57.7	39.7	9.0
South South	12.8	5.9	4.0	61.5	33.7	8.4
South West	12.9	4.6	7.4	32.3	30.0	15.2
Tribe						
Hausa/Fulani	*28.7	*20.5	*21.4	*55.5	*47.2	*29.2
Igbo	6.9	2.0	1.0	56.4	33.7	10.8
Yoruba	6.7	3.4	2.8	30.2	26.3	11.7
Others	18.2	9.7	9.6	63.4	45.3	16.9
Religion						
Islam	*26.9	*18.7	*19.3	56.1	*47.0	*27.3
Christian	12.4	5.6	5.6	56.7	38.4	13.1
Others	22.9	11.4	8.6	61.1	42.9	11.1
Employment						
Employed	22.2	14.4	14.7	56.6	44.4	22.5
Unemployed	18.2	10.5	11.7	50.0	35.5	18.4
Total	22.0	14.3	14.6	56.4	44.1	22.4

*Significant at 5% CM/LWSP Currently married or Living with sexual partner. Ed Education.

In Table 3 and Figure 1, nearly half (48.8%) of the non-users did not go for ANC services because the providers were far from them. This varied significantly with their locations, 33.7% in urban and 52.0% in rural areas. This problem was significantly associated with lower educational attainment, poorer wealth status, Northern zones, ethnicity and employment status of the respondents. About one fifth (21.4%) did not go for ANC because “the service providers were of the opposite sex”. This problem varied significantly with the social-demographic characteristics of the nonusers (except employment status). Of the facility related factors, 25.5% did not attend ANC clinic because the clinics did not have skilled health workers, 24.1% because of poor attitudes of the workers, 20.0% because the non-users could not trust the workers with their personal information, and 22.6% because the facilities did not have good drugs for the attendees.

**Table 3:** **Distribution of service-related reasons for not utilizing ANC services by respondents’ socio-demographic characteristics**

**Characteristics**	**Distance from health facility**	**Different sex with provider**	**No skilled health worker at the facility**	**Poor attitude of the provider**	**Cannot trust provider with personal information**	**Good drugs not available at facility**
Location						
Urban	*33.7	*18.0	22.3	22.8	16.4	20.4
Rural	52.0	22.2	26.2	24.4	20.8	23.1
Education						
No Formal Ed	*53.5	*25.6	*29.1	*27.2	*23.5	*25.8
Qur’anic Only	57.3	27.3	30.4	28.5	24.1	24.7
Primary	41.0	13.9	19.5	18.2	13.6	19.1
Secondary	34.4	10.7	16.3	15.1	11.5	13.0
Higher	38.1	12.2	17.1	31.0	11.9	19.0
Marital Status						
CM or LWSP	49.2	*22.5	26.1	24.7	*20.7	*23.5
Form Married	43.8	7.4	18.9	16.8	11.5	9.5
Never Married	45.6	8.8	19.3	15.8	10.5	14.0
Age At Birth						
<20	53.8	*24.7	25.3	25.3	20.3	22.0
20-24	53.2	23.1	27.5	24.6	21.0	23.5
25-34	48.2	22.9	26.2	25.7	21.2	24.5
35-49	44.7	17.0	22.9	20.9	17.3	19.0
Wealth quintiles						
Poorest	*58.7	*27.0	*30.8	*28.4	*24.9	*27.0
Poorer	49.2	21.9	26.7	24.2	20.7	23.7
Average	40.4	13.4	20.1	18.8	12.5	16.0
Wealthier	28.4	10.6	11.5	14.4	11.1	13.5
Wealthiest	20.9	14.3	15.2	20.7	12.1	14.1
Zone						
North Central	*49.2	*14.3	*19.4	*21.3	*15.0	*13.7
North East	63.5	32.0	43.0	35.4	28.1	40.8
North West	51.1	28.7	28.8	28.1	26.1	24.9
South East	40.3	3.8	13.0	10.4	9.1	12.8
South South	36.4	6.2	11.0	9.9	7.7	11.7
South West	30.9	8.8	12.9	14.3	7.4	10.6
Tribe						
Hausa/Fulani	*53.4	*29.6	*30.5	*29.3	*26.4	*26.6
Igbo	36.3	3.9	13.7	12.7	8.9	15.8
Yoruba	27.8	7.8	11.2	14.5	8.9	11.7
Others	48.8	15.5	23.4	20.6	15.1	20.5
Religion						
Islam	*52.1	*27.7	*30.0	*28.7	*24.3	*27.0
Christian	41.8	9.6	17.0	15.5	12.0	14.4
Others	54.3	8.6	16.7	14.3	11.4	11.1
Employment						
Employed	*49.1	21.6	25.5	24.1	20.0	22.6
Unemployed	39.5	15.8	26.3	22.4	18.4	22.4
Total	48.8	21.4	25.5	24.1	20.0	22.6

*Significant at 5% CM/LWSP Currently married or Living with sexual partner.

**Figure 2:**
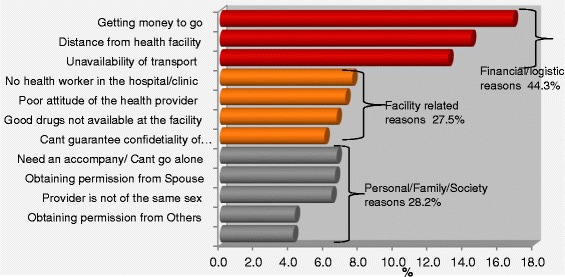
Contributions of each reason to all reasons given for not attending ANC.

The analysis of multiple responses presented in Table 4 showed that of the 2199 non-users, 55.7% claimed they had problems getting money to go to ANC facility, 48.2% reported having problem with the distance to the health facilities while 43.6% did not go for ANC because there wasn’t any means of transportation to the facilities. Among all the reasons given for not attending ANC services, “Getting money to go” ranked single most important reason, been highest with 16.8% of all the problems, followed by “Distance from health facility” 14.4% and “Availability of transport to the facilities” 13.1%. These three reasons jointly constituted nearly half (44.3%) of all the reasons why the respondents did not use the ANC facilities. Health facility related factors comprising of unavailability of good drugs and skilled health workers, poor attitude and unprofessional conduct of the health workers made up 27.5% of the reasons why the pregnant women did not attend ANC services. The remaining 28.1% of the reasons for not attending ANC services were individual differences, family and societal reasons as shown in Figure 2. Elimination of the three most important problems would increase ANC coverage by about 15% while overcoming health facility loopholes could further boost ANC coverage by almost 9%.

**Table 4:** **Analysis of multiple reasons for not using ANC services**

**Reasons**	**N**	**% who gave each reason (95% CI)**	**% among all reasons(95% CI)**
Getting money to go	1225	56.4(53.6-57.8)	16.8(15.9-17.6)
Distance from health facility	1060	48.8(46.1-50.3)	14.5(13.7-15.3)
Availability of transport	959	44.6(41.5-45.7)	13.1(12.3-13.9)
No health worker in the hospital/clinic	555	25.5(23.4-27.1)	7.6(7.0-8.2)
Poor attitude of the health provider	524	24.1(22.0-25.6)	7.2(6.6-7.8)
Need an accompany/Can not go alone	486	22.4(20.4-23.8)	6.7(6.1-7.2)
Good drugs not available at the facility	491	22.6(20.6-24.1)	6.7(6.1-7.3)
Obtaining permission from Spouse	479	22.0(20.1-23.5)	6.6(6.0-7.1)
Provider is not of the same sex	466	21.4(19.5-22.9)	6.4(5.8-6.9)
Can not guarantee confidentiality of information	435	20.0(18.1-21.4)	6.0(5.4-6.5)
Obtaining permission from Others	318	14.6(13.0-15.9)	4.4(3.9-4.8)
Obtaining permission from Guardian/Parent	310	14.3(12.6-15.6)	4.2(3.8-4.7)

CI Confidence Interval.

## Discussions

We determined and prioritized the reasons why non-users of ANC chose not to access services in Nigeria. The highest non-users of ANC were found among the poor, rural, currently married, and less educated respondents from the Northern part of Nigeria, especially in the North East zone. Significant associations were found between the stated reasons for non-utilization of ANC among the respondents and socio-demographic characteristics. The reasons varied significantly with regards to respondents’ economic status, educational attainment, residence, geographical locations, age and marital status. Our analysis revealed that some respondents with higher educational attainment, and also in the wealthiest quintiles, did not use ANC services during their last pregnancy. This indicated that ANC utilization was not only influenced by poverty and lack of information but also by other factors, such as seeking permissions from spouses and partners, beliefs, dispositions and views on quality and attitudes towards the ANC providers, and distances to health facilities. We found that three reasons were central to non-utilization of ANC services in Nigeria: “Problems with getting money to go health facilities”, “Farness of ANC service providers” and “Unavailability of transport to reach the ANC providers”.

These three problems concern finance and logistics, and they collectively constituted nearly half of all the reasons why pregnant women did not use the ANC services. Therefore, the ANC coverage of 65.1% in Nigeria according to NARHS 2012 [1] might increase by 15.4% to over 80% if more ANC facilities were established and ANC services were made totally free across Nigeria. Of all the identified barriers to ANC utilization, inability to pay for the services was the most common problem preventing pregnant women from accessing the ANC in Nigeria. This finding is consistent with previous findings [2,9,20-22,24,28]. United Nations reported that poverty is a major barrier to ANC utilization across developing countries [29]. A recent Zambia study [30] also found a strong connection between distance to a health facility, the ANC usage and the quality of ANC received.

Previous studies have recommended that for the ANC coverage in developing countries to match the coverage in most developed countries, the ANC services should be made free and available especially in rural settings [2,21,23,29], with at least one ANC facility within every 15 km radius [30,31] and staffed with various healthcare professionals [2,30,32,33].

Factors related to health facilities included lack of good medications and skilled health workers. Poor attitude and unprofessional conduct of health workers made up over a quarter of the reasons why pregnant women did not utilize the ANC services. Other studies ascribed such findings to the fact that the previous personal experiences with ANC facility staff, or experiences narrated by women’s friends or family members, may affect the care-seeking behavior [12,23,30,34]. Additional ANC coverage of 9.6% could be achieved if all existing health facilities were supplied with adequate drugs and manned by skilled health workers who exhibited professional attitudes and behaviours.

We also identified that personal, family, societal and cultural factors affect utilization of ANC, this is in agreement with outcomes of a meta-analysis of qualitative studies reporting the views and experiences of a total of 1,230 women in 15 low and middle income countries who received inadequate ANC [28]. Authors of the latter concluded that any ANC programming at odds with both local theoretical and contextual beliefs and experiences may suffer under-utilization. Obtaining permission from spouses, family members, in-laws, society leaders to visit ANC facilities remains a problem in Nigeria [6]. Our findings were corroborated with the report entitled “Antenatal Care: Opportunities for Africa’s Newborns” [2] which highlighted the need to establish meaningful contact between communities and facilities in order to increase utilization of ANC. In North Nigeria, especially the Eastern part, Islam is the main religion and most husbands practicing Islam forbid their wife going out without outright permission [6,7]. Factors related to family and society have been estimated to result in a nearly 10% loss in ANC coverage in Nigeria. There is need for partners and other family members to embrace the ANC and encourage it among pregnant women as their support can help women follow recommendations offered by the ANC providers, promote joint decision making among partners, and improve the health of mothers and newborns.

### Study limitations

We used a secondary data which relied solely on ability of respondents to correctly recall and report past events without any means of further verification by the interviewers. The data might have suffered recall bias and other non-sampling errors. Data on some variables that could have been of interest to the researchers were not collected.

## Conclusions

Affordability, availability, and accessibility of ANC providers are the most common problems facing utilization of ANC in Nigeria. Poor, rural women with limited education in particular face challenges in these. Joint efforts should be deployed to making ANC services attractive to and reachable by pregnant women and nursing mothers. These efforts should address financial and cultural barriers to ANC use, quality improvement to increase ANC services utilization and satisfaction, and maximal contacts between the woman, the service providers and the health services.

Implementation of a free ANC policy, the establishment of more ANC public health facilities within a 15 km radius of every woman across Nigeria, and an emphasized focus on the WHO public health guidelines on ANC are the surest ways to overturn the low ANC coverage in Nigeria. The health facilities should be supplied with adequate drugs, manned by skilled health workers and the workers re-orientated to be professionals so as to win confidence and patronage of women and their partners. Priority must be given to recruitment and to efforts in retaining skilled health workers, and to their adequate supervision, training, knowledge and skills acquisition, and motivation in addition to establishment of health facilities, availability of drugs, equipment and other consumables.
